# Relationship between endophenotype and phenotype in ADHD

**DOI:** 10.1186/1744-9081-4-4

**Published:** 2008-01-30

**Authors:** Nanda NJ Rommelse, Marieke E Altink, Neilson C Martin, Cathelijne JM Buschgens, Stephen V Faraone, Jan K Buitelaar, Joseph A Sergeant, Jaap Oosterlaan

**Affiliations:** 1 Department of Clinical Neuropsychology, VU University Amsterdam, Van der Boechorststraat 1, 1081 BT Amsterdam, The Netherlands; 2 Department of Psychiatry, Radboud University Nijmegen Medical Center, Reinier Postlaan 10, 6500 HB Nijmegen, The Netherlands; 3 School of Psychology, Curtin University of Technology, Kent Street, Bentley, Perth 6845, Western Australia; 4 Departments of Psychiatry and Neuroscience & Physiology, SUNY Upstate Medical University, 301 713 Harrison Street, Syracuse, NY 13210, USA

## Abstract

**Background:**

It has been hypothesized that genetic and environmental factors relate to psychiatric disorders through the effect of intermediating, vulnerability traits called endophenotypes. The study had a threefold aim: to examine the predictive validity of an endophenotypic construct for the ADHD diagnosis, to test whether the magnitude of group differences at the endophenotypic and phenotypic level is comparable, and to investigate whether four factors (gender, age, IQ, rater bias) have an effect (moderation or mediation) on the relation between endophenotype and phenotype.

**Methods:**

Ten neurocognitive tasks were administered to 143 children with ADHD, 68 non-affected siblings, and 120 control children (first-borns) and 132 children with ADHD, 78 non-affected siblings, and 113 controls (second-borns) (5 – 19 years). The task measures have been investigated previously for their endophenotypic viability and were combined to one component which was labeled 'the endophenotypic construct': one measure representative of endophenotypic functioning across several domains of functioning.

**Results:**

The endophenotypic construct classified children with moderate accuracy (about 50% for each of the three groups). Non-affected children differed as much from controls at the endophenotypic as at the phenotypic level, but affected children displayed a more severe phenotype than endophenotype. Although a potentially moderating effect (age) and several mediating effects (gender, age, IQ) were found affecting the relation between endophenotypic construct and phenotype, none of the effects studied could account for the finding that affected children had a more severe phenotype than endophenotype.

**Conclusion:**

Endophenotypic functioning is moderately predictive of the ADHD diagnosis, though findings suggest substantial overlap exists between endophenotypic functioning in the groups of affected children, non-affected siblings, and controls. Results suggest other factors may be crucial and aggravate the ADHD symptoms in affected children.

## Background

**Figure 1:**
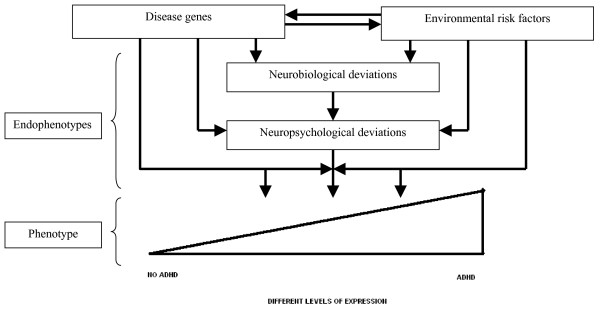
The relationship between genetic and environmental risk factors, endophenotypes, and phenotype in ADHD.

Psychiatric disorders as defined by the Diagnostic and Statistical Manual of Mental Disorders (DSM-IV-TR) [1] have been hypothesized as reflecting the extreme end of underlying, continuously distributed traits [2-4]. In line with this, the behaviour of individuals suffering from psychiatric disorders differs quantitatively but not necessarily qualitatively from the behaviour of individuals without psychiatric problems. The threshold of what is and what is not abnormal is to a certain extent arbitrarily determined, but patients have in common that their behaviour interferes with their normal life and cause the patient (and his/her environment) to suffer. Why certain people pass this threshold and are diagnosed with a disorder and others do not is determined by additive and interacting genetic and environmental risk factors [5,6]. Studies have shown that psychiatric disorders have genetic and environmental underpinnings which probably contribute to certain neurocognitive abnormalities that, in turn, lead to abnormal behaviour. It is theorized that these neurocognitive abnormalities form underlying, continuously distributed, vulnerability traits (endophenotypes) that heighten the risk for developing a disorder (phenotype) [7-9]. In this context, neurocognitive abnormalities refer to mental functions that are mediated by brain processes; these mental functions are not directly observable, but may be manipulated and measured using experimental paradigms. Multiple endophenotypes interact to determine the finally observable behavior, the phenotype, which might be abnormal. In this context, the phenotype refers to directly observable symptoms of a disorder (Figure 1).

Studying endophenotypes may have certain advantages over studying phenotypes. It has been proposed that endophenotypes may be more suitable for detecting risk genes, because endophenotypes are genetically less complex than phenotypes (i.e. related to fewer genes than phenotypes) and, therefore, probably stronger linked to these disease genes than phenotypes [7-9]. Endophenotypes may also be useful in exploring different pathways leading up to the disorder: Patients having the same diagnosis may differ strongly in the number and severity of symptoms they portray, suggesting heterogeneity in the causal pathways [10]. Creating more homogeneous subgroups of patients based on their endophenotypic functioning, may facilitate unravelling these differential causal pathways.

In the last two decades, substantial attention has been given to studying endophenotypes of psychiatric disorders. This has led to the discussion of what exactly constitutes an endophenotype and what criteria must be met for a neurocognitive function to be useful as candidate endophenotype [7-9,11-14]. Since an endophenotype forms a link between susceptibility genes and the disorder, it follows that: (1) the neurocognitive dysfunction is heritable (and familial), in which at least partly the same genes influence the endophenotype and phenotype; (2) the neurocognitive dysfunction is associated with the disorder; (3) the neurocognitive dysfunction is observable in non-affected first-degree relatives of an affected individual, because first-degree relatives are likely to carry some of the susceptibility genes of the disorder.

Thus far, several psychiatric conditions have been targeted for candidate endophenotypes and considerable knowledge has been gathered on the usefulness of different endophenotypes in for example, bipolar disorder, schizophrenia, substance abuse, and depression [15-19]. This study targeted Attention-Deficit/Hyperactivity Disorder (ADHD), since it is one of the most prevalent child psychiatric disorders, yet the knowledge on the usefulness of ADHD endophenotypes is still limited.

Two studies have failed to find neurocognitive impairments in parents of children with ADHD [20,21] and two studies found no conclusive evidence of cognitive dysfunctioning in non-affected siblings [22,23]. However, one study did find evidence for cognitive functions as endophenotypes [24] and other studies, specifically targeting inhibition or interference control, found evidence for these functions as endophenotypes [13,25-28]. In a previous study, we also found evidence for inhibition as well as visuo-spatial and verbal working memory as endophenotypes for ADHD [29]. Moreover, it appeared that deficits in the various cognitive functions partly arose from the same genetic and/or environmental risk factors. Furthermore, evidence has been found for time reproduction as ADHD endophenotype, a function related to the sense of time [30]. Less attention has been given to studying functions outside the cognitive domain, although ADHD is frequently associated with motor deficits in Caucasian and non-Caucasian subjects [31,32]. In previous studies, we and others have shown that non-affected siblings have subtle problems, similar to their affected siblings, in motor timing, motor control, motor speed and variability, and speed of oculomotor control [33-36], suggesting endophenotypes for ADHD may also lie inside the motor domain. Moreover, it appeared that some of the cognitive dysfunctioning in children with ADHD and their non-affected siblings may be related to problems already apparent on a simple reaction time task [31].

Taken together, the majority of studies have found support for cognitive and motor endophenotypes for ADHD [24,26,28-31,33-35]. These studies have in common that they administered one or a few measures tapping into a single domain. It is unlikely, however, that one such measure/domain mediates the relation between genotype and phenotype and can predict the phenotype, because it is unlikely that all children with ADHD and their non-affected siblings will show this endophenotype given the causal heterogeneity of ADHD [13]. It is more likely that multiple endophenotypes mediate the relation between genotype and phenotype, and together are more powerful in predicting the phenotype. Therefore, the first aim of our study was to investigate if an endophenotypic construct, encompassing a broad battery of both cognitive and motor endophenotypes, is predictive of the ADHD diagnosis.

Practically all studies of ADHD endophenotypes conducted thus far have reported the same type of results: The group of non-affected siblings performs in between the affected siblings group and the normal control group. At a phenotypic level, non-affected siblings do not (or apparently to a lesser extent) deviate from the controls. The reverse appears to be true for affected children: Their phenotypic deficits are more pronounced than one would expect based on their cognitive and motor dysfunctioning. Therefore, the second aim of the paper was to confirm apparent observations that children with ADHD show more severe ADHD symptoms than one would expect based on their endophenotypic dysfunctioning, whereas non-affected siblings show less ADHD-like behaviour than one would expect based on their endophenotypic vulnerabilities.

The question that automatically arises if these observations can be confirmed, is why endophenotypic vulnerabilities are not proportionally related to deviations at a phenotypic level? It might be that certain factors moderate and/or mediate the relation between endophenotype and phenotype. Moderation would imply that the relation between endophenotype and phenotype is not comparable across different levels of the moderating factor [37]. Mediation would imply that (a part of) the relation between endophenotype and phenotype can be explained through their correlation with a third factor. When this third factor is taken into account, the relation between endophenotype and phenotype disappears or weakens [37]. The third aim of this study was to explore whether four factors (gender, age, IQ, and rater bias) had a (moderating and/or mediating) effect on the relationship between endophenotype and phenotype.

With respect to the first factor gender: ADHD is more frequently diagnosed in boys than girls [38], probably because boys are more vulnerable to the disorder. Since affected children are more often boys than girls, whereas the gender ratio is more or less equal in the group of non-affected siblings, moderating and/or mediating effects of gender may possibly be accountable for the apparent non-comparable magnitude of group differences at the endophenotypic and phenotypic level. With respect to the second factor age: It is known from several studies that the severity of ADHD symptoms appears to decline to some extent with age [39-41] and that the same might be true for underlying neurocognitive vulnerabilities [30]. Yet, others have failed to find diminishing neurocognitive vulnerabilities with age [40,42,43]. It may thus be possible that age has a moderating and/or mediating influence on the relation between endophenotype and phenotype. With respect to the third factor IQ: A frequently reported finding is that children with ADHD have on average a lower IQ than controls [44-46]. It has been suggested that this lower IQ may underlie cognitive dysfunctioning, or vice versa, that cognitive dysfunctioning is at the heart of a lower intelligence, or that there is no hierarchical relation between both domains but both domains share common causes [47-49]. Either way, since IQ seems both associated with the neurocognitive dysfunctioning as well as with ADHD behaviour, a mediating effect of IQ may be expected on the relation between neurocognitive dysfunctioning and ADHD.

An additional factor that may influence the relation between endophenotype and phenotype may be rater bias: Parents may underestimate the severity of ADHD symptoms in their undiagnosed/non-affected child and overestimate the severity of ADHD symptoms in their diagnosed/affected child. Teachers, however, may be less likely to be affected by this contrast effect, since they often do not have siblings of the same family in their class due to age differences between the siblings. Rater bias explaining the non-comparable magnitude of group differences at the endophenotypic and phenotypic level may thus be investigated by comparing the relation between endophenotype and phenotype as observed by parents and teachers.

The first aim was to investigate if an endophenotypic construct, encompassing a broad battery of both cognitive and motor endophenotypes, is predictive of the ADHD diagnosis. The second aim was to confirm apparent observations that children with ADHD show more severe ADHD symptoms than one would expect based on their endophenotypic dysfunctioning, whereas non-affected siblings show less ADHD-like behaviour than one would expect based on their endophenotypic vulnerabilities. The third aim of this study was to explore the (moderating and mediating) effects of four factors on the relationship between endophenotype and phenotype: gender, age, IQ, and rater bias.

## Methods

### Sample

Families with at least one child with the combined subtype of ADHD (proband) and at least one additional sibling (regardless of possible ADHD-status) were recruited in order to participate in the Dutch part of the International Multicenter ADHD Genes study (IMAGE). The IMAGE project is an international collaborative study that aims to identify genes that increase the risk for ADHD using QTL linkage and association strategies [50]. Additional control families were recruited from primary and high schools in the same geographical regions as the participating ADHD-families. Controls and their first degree relatives were required to have no formal or suspected ADHD diagnosis. For this study, we selected a subsample of first- and second-borns to rule out dependency of the data. In the first-borns, 143 affected children, 68 non-affected siblings and 120 controls participated. In the second-borns, 132 affected children, 78 non-affected siblings, and 113 controls participated. All children were between the ages of 5 and 19 years and were of European Caucasian descent. Participants were excluded, if they had an IQ < 70, a diagnosis of autism, epilepsy, general learning difficulties, brain disorders or known genetic disorders, such as Down syndrome or Fragile-X-syndrome.

The screening procedures and measures for phenotyping have been described elsewhere [50]. Briefly, screening questionnaires (parent and teacher Conners' long version rating scales [51] parent and teacher Strengths and Difficulties Questionnaires [52] and parent Social Communication Questionnaire [53]) were used to identify children with ADHD symptoms and to screen for any autistic like behaviours. Scores were indicative for a diagnosis of ADHD if *T*-scores were ≥ 63 on the Conners' ADHD-subscales (DSM-IV Inattention, DSM-IV Hyperactive-Impulsive, and DSM-IV ADHD Total) and > 90^th ^percentile on the SDQ-Hyperactivity scale. A score of ≥ 15 on the SCQ was considered indicative of autistic like behaviours. Parents and teachers were asked to rate the behavior of the child when off medication. Concerning all children rated clinically on any of the questionnaires completed either by parents or teachers, the Parental Account of Children's Symptoms (PACS) was administered [54]. Data from the questionnaires and the PACS were subjected to a standardised algorithm to derive each of the 18 DSM-IV ADHD symptoms, providing operational definitions for each behavioural symptom [30]. With respect to control children, the Conners' long version for both parents and teachers was completed and all control children were required to obtain non-clinical scores.

Full-scale IQ was estimated by four subtests of the Wechsler Intelligence Scale for Children III (WISC-III) or Wechsler Adult Intelligence Scale III (WAIS-III) (depending on the child's age): Vocabulary, Similarities, Block design and Picture completion [55,56]. These subtests are known to correlate between .90–.95 with the Full-scale IQ [57]. IQ testing took place while the children were off medication.

### Procedure

Testing of children with ADHD and their siblings took place at the VU University Amsterdam or at the Radboud University Nijmegen Medical Centre and was conducted simultaneously for all children in a family. Psychostimulants were discontinued for at least 48 hours before testing took place [58]. Children were motivated with small breaks. At the end of the session, a gift worth approximately € 4, – was given. Control children were tested in a similar way in a quiet room at their school. The study had medical-ethical approval by the local ethics committee and was in accordance with the Helsinki Declaration.

### Experimental tasks

The ten experimental tasks described in this study have been fully described elsewhere [29-31,33,34]. A short description of each task will be given below. Based on previous results [29-31,33,34], the variable per task that showed most optimal results in the endophenotypic analyses in the five previous studies was chosen for the current analyses.

#### Stop task

The Stop task was used to measure speed of inhibition of an ongoing response [59,60]. Subjects were presented two types of trials: go-trials and stop-trials. Go-trials consisted of the presentation of a go-stimulus (drawing of a plane) that was either pointing to the right or to the left [61]. Children were instructed to press a response button that corresponded to the direction of the stimulus as quickly and as accurately as possible. Stop-trials were identical to the go-stimulus but in addition a stop-signal was presented (drawing of a cross that was superimposed on the plane). Children were required to withhold their response to the stop-signal. Go stimuli were displayed for 1000 ms, preceded by a 500 ms fixation point. Stop signals were displayed for 1000 ms minus delay time, with a mean display time of 709 ms (*SD *125 ms). Inter-trial intervals were 3000 ms. The delay between the go- and stop-signal was dynamically varied so that it could be estimated when the child successfully inhibited 50% of the stop-trials, and unsuccessfully inhibited the other 50%. At this point, the go-process and stop-process were of equal duration, which made it possible to estimate the latency of the stop-process: the Stop signal reaction time (SSRT) [59]. A total of 2 practice blocks and 4 experimental blocks were administered, each consisting of 60 trials. The first practice block consisted of only go-trials. The second practice block and the 4 experimental blocks consisted of 75% go-trials and 25% stop-trials. Go- and stop-trials were pseudo-randomly presented. Task administration took about 15 min. Based on previous results, the dependent measure was the SSRT [29], which showed endophenotypic-like group differences and correlated between siblings.

#### Shifting attentional set

Shifting attentional set was designed to measure accuracy of motor inhibition and cognitive flexibility [62]. The task consisted of three blocks of which the first block was designed to acquire a baseline of the accuracy of responding with which the performance on the second (motor inhibition) and third (cognitive flexibility) block could be compared. In all blocks, trials consisted of a horizontal bar with ten grey squares presented permanently at the centre of the screen. From trial to trial, a coloured square moved across the bar in a random direction (either one square to the right or to the left). Responses were required to be initiated between 150 to 5000 ms after a square moved one position, otherwise a trial was replaced. The task was self-paced with post-response intervals of 250 ms. In the first block, the moving square was coloured green, and compatible responses were required: children were instructed to press a response button as quickly and as accurately as possible that corresponded to the direction in which the stimulus moved. In the second block, the moving square was coloured red, and incompatible responses were required. The suppression of the automatic compatible response, in order to generate a non-automatic incompatible response, was hypothesized as requiring inhibitory control. In the third block, the colour of the moving square alternated randomly between green and red, and both compatible and incompatible responses were required. Thus, both the direction and the colour of the square were unpredictable. The mixture of both compatible and incompatible trials was hypothesized to require high levels of cognitive flexibility in addition to inhibitory control [63]. The first and second block consisted of 10 practice trials and 40 experimental trials. The third block consisted of 16 practice trials and 80 experimental trials. Administration took about 10 to 15 min. The dependent measure was the percentage of errors across blocks, which was the best indicator of endophenotypic vulnerabilities [31].

#### Time reproduction

The Timetest application version 1.0 [64] was used to measure time reproduction. Stimuli consisted of temporal intervals with different durations (4, 8, 12, 16, 20 s) that had to be reproduced as accurately as possible. The task was administered first in the visual modality (light bulb) and thereafter in the auditory modality (tone). Children were not informed about the length of the intervals. In both modalities, 3 practice and 20 experimental trials were administered. The five interval lengths were randomly presented four times. Task administration for both modalities required 15 min. Based on previous results, the main dependent measure was the precision of the reproduction (operationalized as the absolute discrepancy between the response length and the stimulus length) averaged across trials and modalities, which was abnormal in children with ADHD and their non-affected siblings and correlated between siblings [30].

#### Visuo-spatial sequencing

The Visuo-spatial sequencing task was used to measure accuracy of visuo-spatial working memory [62]. Stimuli consisted of nine circles symmetrically organized in a square (3 by 3). On each trial, a sequence of circles was pointed at by a computer-driven hand. Subjects were instructed to replicate the exact same sequence of circles, by pointing to them with the small, self-driven hand. There were no time constrictions. One practice trial and 24 experimental trials were presented. Every succeeding trial increased in difficulty level: an increase in the number of circles required to be remembered and/or an increase in the complexity of the spatial pattern (i.e. the trial consisted of circles that were spatially further removed from one another instead of being close to one another), hence manipulating working memory demands. Task administration took about 7 min. Based on previous results, the total number of correct targets in the correct order was used as dependent measure reflecting endophenotypic-like group differences and correlating between siblings [29].

#### Digit span

The Digit span backwards of the WISC-III and WAIS-III [55,56] was used to obtain an indication of verbal working memory. The backward part consisted of repeating a sequence of numbers in the opposite order. Children were instructed to reproduce sequences as accurately as possible. One digit was added to the sequence if a child reproduced the sequence successfully. Two practice trials with a 2 digit sequence and (dependent on the child's performance) a maximum of 8 experimental sequences were administered. Dependent measure was the maximum Digit span backwards, which proved useful as endophenotypic candidate [29].

#### Pursuit

This task was designed to measure precision of motor control under continuous adaptation [62]. The stimulus consisted of a randomly moving target (asterisk) that was required to be 'caught' by moving a mouse cursor on top of the asterisk. The target moved at a constant speed of 10 mm/s. Children were instructed to 'catch' the randomly moving target as precisely as possible. One practice (13 s) and one experimental session (60 s) were administered for both hands separately. Administration took about 5 min. The dependent measure was the precision (mean distance in mm between target and cursor calculated per second and averaged across the 60 s experimental session) of the left hand. Previous results have shown that mainly the performance of the non-dominant hand was most strongly associated with ADHD [33].

#### Tracking

This task aimed to measure precision of motor control without continuous adaptation required [62]. The stimulus consisted of an inner and outer circle (radius 7.5 and 8.5 cm, respectively). Children were instructed to trace an invisible midline (radius 8 cm) between the inner and outer circle as quickly and precisely as possible with a mouse cursor. One practice and one experimental session were administered for both hands separately (clockwise with the right hand and counter clockwise with the left hand). Administration took about 3 min. The dependent measure was the precision (mean distance to midline in mm averaged across 60 equal parts of the circle) of the left hand. Previous results have shown that precision of the non-dominant hand showed endophenotypic-like characteristics [33].

#### Tapping

This task measured variability of self-generated motor output [62]. This task required the child to tap as frequently as possible within a certain time period. During tapping, the number of taps was continuously counted and displayed on the screen. One practice session (5 s) and one experimental session (18 s) were administered for both hands separately. The task was first practised and executed with the index finger of the non-preferred hand, thereafter practised and executed with the index finger of the preferred hand. Administration took about 3 min. The dependent measure was the variability (SD of intertap intervals in ms) averaged across hands. Previous results have shown that this measure correlates between siblings [34].

#### Baseline speed

This task was designed to measure variability on a simple reaction time task [62]. Stimuli consisted of a fixation cross in the centre of a computer screen that changed unpredictably into a white square. Immediately following the response, the white square changed back into the fixation cross. The time interval between a response and the emergence of the next white square varied randomly between 500 to 2500 ms in order to prevent anticipation strategies. Subjects were required to press a key as quickly as possible when the white square appeared. A practice session (10 trials) and an experimental session (32 trials) were administered for both hands separately. The task was first practised and executed with the index finger of the non-preferred hand, thereafter practised and executed with the index finger of the preferred hand. Administration took about 5 min. Dependent measure was the variability (SD of reaction times in ms) of responses averaged across hands. Previous results have shown that this measure was associated with ADHD and correlated between siblings [34].

#### Motor timing

This task was designed to measure variability of motor timing [65]. In this task a 1 s interval had to be produced. The start of the interval was announced by a tone (80 db, 50 ms). After the subject's response, visual feedback was given, indicating whether the response was correct, too short or too long. A response was regarded as correct, if it fell between the lower and upper boundary set by a dynamic tracking algorithm. Boundaries were set at 500 to 1500 ms at the beginning of the task. If the response fell within these boundaries, the boundaries for the subsequent trial were narrowed by 100 ms. Likewise, the boundaries of the subsequent trial were widened with 100 ms, if the response on the previous trial fell outside those boundaries. Subjects were instructed to produce as accurately as possible the 1 s interval. Twenty practice trials and 80 experimental trials were administered. Both sessions were preceded by presenting 10 times a cartoon figure for exactly 1 s on the screen to demonstrate the duration of 1 s [65]. Administration took about 8 min. The dependent measure was the variability (*SD *of productions in ms). Previous results have shown that this measure to be a viable endophenotypic candidate [34].

### Data analyses

Missing data for all variables for the sample described in this study was less than 5% and were replaced by means of expectation maximization [66]. The task measures were successfully normalized and standardized to *z*-scores by applying a Van der Waerden transformation (SPSS version 14). Some of the *z*-scores were multiplied by -1, so that the *z*-scores of all task variables would have the same meaning: A higher *z*-score was indicative of poor performance or underestimation. Correction for multiple comparisons according to the false discovery rate (FDR) controlling procedure was applied to the analyses with a q-value setting of 0.05 [67].

In order to rule out dependency of the data (more than one child per family participated in the study), analyses were performed with the children split by birth order. First, analyses were run on the data from the first-borns (*N *= 331), thereafter repeated on the second-born children (*N *= 323). In this way, not only was the dependency of data handled, but this also gave the opportunity to study replicability of the results. Since most families consisted of two children, the samples of third- and fourth-borns were substantially smaller (*N *= 76 and *N *= 11, respectively) than the samples of first- and second-borns. These sample ratio differences were considered too large (> 4) and therefore, the third- and fourth-borns were excluded from the analyses.

To combine the task variables to a component that would simplify the analyses and reduce error variance, a principal component analysis was performed on the ten task variables, separately for the first- and second-borns. The component explaining the largest amount of variance was used in the further analyses and is labelled 'the endophenotypic construct'. The standardized Conners' ADHD total raw score was averaged across parents and teachers and labelled 'the phenotype'.

The first aim of this paper was to investigate whether the endophenotypic construct was predictive of the ADHD diagnosis (affected, non-affected, control) using a multiple discriminant analysis. Age was also entered in the model, because age differences had arisen between the first- and second-borns by splitting up the sample. There was assessed what percentage of children was correctly classified by the endophenotypic construct.

The second aim of this paper was to test whether children with ADHD showed a more severe phenotype than one would expect based on their endophenotypical dysfunctioning and whether the reverse was true for the non-affected siblings. Therefore, the interaction of group (affected, non-affected, control) by functioning (2 within subject levels: endophenotypic construct and phenotype) was tested in a repeated measures ANOVA. Age was again implemented as covariate.

**Figure 2:**
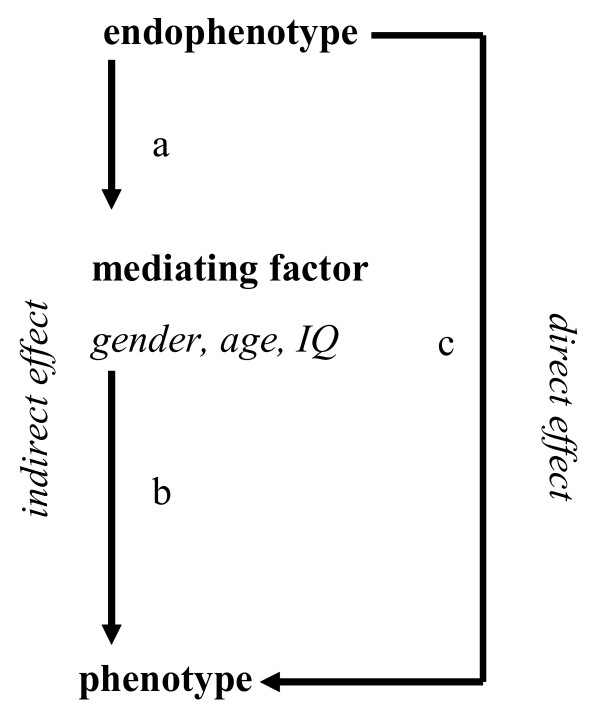
Display of possible mediating effects of gender, age, and IQ between endophenotype and phenotype.

The third aim of this paper was to investigate whether the factors gender, age, and IQ may moderate and/or mediate the relation between the endophenotypic construct and the phenotype. Using regression analyses, significant moderation for a factor would be demonstrated if the predictive effect of the endophenotypic construct was not constant for that factor and was tested by examining the interaction between the endophenotypic construct and the factor in predicting the phenotype [37]. Mediation was tested by calculating the path coefficients between (a) the endophenotypic construct and the factor, (b) the factor and the phenotype, and (c) the endophenotypic construct and phenotype (see Figure 2). The effect of mediation (in which endophenotypical dysfunctioning lead to ADHD through gender, age, and/or IQ) is termed the indirect effect [36] and can be tested using the formula [68]: z-value = *a *× *b*/√(*b*^2^*s*_*a*_^2 ^+ *a*^2^*s*_*b*_^2^), in which *a *represents the unstandardized path coefficient of the endophenotypic construct on the mediating factor, *b *represents the unstandardized path coefficient of the mediating factor on the phenotype, *s*_*a*_^2 ^and *s*_*b*_^2 ^represent the square of the standard error of the path coefficients *a *and *b*. It was examined whether the direct effect between the endophenotypic construct and phenotype was still significant after controlling for the indirect effect.

Rater bias could not be examined for moderation and mediation, since the phenotype was confounded with the moderating or mediating factor. Therefore, a different approach was taken. Paired sample *t*-tests were used to compare parental and teacher ratings of ADHD to assess whether the phenotype differed when rated by parents or teachers. Additionally, in order to investigate whether the predictive validity of the endophenotypic construct was different for the phenotype as rated by the parents or by the teachers, the correlation coefficients between the endophenotypic construct – phenotype rated by parents on the one hand and the endophenotypic construct – phenotype rated by teachers on the other hand were compared using two-sided dependent samples *t*-tests [69].

## Results

The percentage of affected, non-affected and controls was equally distributed across the first- and second-borns (χ^2 ^= 1.24, *df *= 2, *p *= .54). The characteristics of both samples are presented in Table 1.

**Table 1:** Sample characteristics

	**first-borns (*N *= 331)**								**second-borns (*N *= 323)**							
	affected		non-affected		control				affected		non-affected		control			
	*N *= 143 (43%)		*N *= 68 (21%)		*N *= 120 (36%)				*N *= 132 (41%)		*N *= 78 (24%)		*N *= 113 (35%)			
	*M*	*SD*	*M*	*SD*	*M*	*SD*	*F*_2,328_	contrasts^1^	*M*	*SD*	*M*	*SD*	*M*	*SD*	*F*_2,320_	contrasts^1^
age in years	13.2	2.6	14.9	2.7	13.3	2.7	10.9	2 > 1 = 3	11.6	2.7	9.7	2.4	10.6	2.9	11.3	1 > 2 = 3
% male	76.9		45.6		44.2		34.8^2^	1 > 2 = 3	75.8		48.7		38.1		37.4^2^	1 > 2 = 3
estimated full scale IQ	99.8	11.8	101.8	10.7	106.2	9.9	11.3	1 = 2 < 3	99.2	11.8	105.5	10.9	105.6	10.1	13.2	1 < 2 = 3
Conners' parent DSM-IV																
inattentive	68.6	10.0	48.6	7.2	46.3	4.5	306.6	1 > 2 = 3	69.7	10.4	46.8	6.5	46.7	4.9	329.9	1 > 2 = 3
hyperactive-impulsive	75.4	12.9	48.4	6.9	47.3	4.9	343.1	1 > 2 = 3	76.0	11.3	49.6	6.8	47.6	5.2	410.0	1 > 2 = 3
Conners' teacher DSM-IV																
inattentive	64.1	9.5	48.8	6.4	46.2	4.7	211.4	1 > 2 = 3	65.7	9.3	48.0	5.6	46.3	4.2	279.0	1 > 2 = 3
hyperactive-impulsive	68.2	11.7	48.5	7.7	46.3	3.9	236.2	1 > 2 = 3	69.0	11.5	47.7	4.7	47.2	5.5	260.5	1 > 2 = 3

*Note*. 1 = affected; 2 = non-affected; 3 = controls. ^1 ^= contrasts based on *p*-values of ≤ .05. ^2 ^= χ^2^.

ADHD = Attention-Deficit/Hyperactivity Disorder; DSM-IV = Diagnostic and Statistical Manual for Mental Disorders (4^th ^edition).

### Principal component analysis

A principal component analysis was performed on the ten task measures, separately for the first- and second-borns. Similar results were obtained in both samples: All ten task measures related to one major component, explaining respectively 40.5% and 47.0% of the variance in the task measures. Additional components did not have an eigenvalue above 1 and explained only 10.3% or less additional variance. Therefore, the following results report only the main component labelled 'the endophenotypic construct'. This endophenotypic construct was representative of endophenotypic functioning across ten tasks, because all tasks loaded on this component score (Table 2). Results were similar, when the analysis was repeated using the raw (not standardized and not normalized) task variables, with a one-component solution explaining 34.9% and 41.5% of the variance in the first- and second-borns respectively, and with all variables correlated with this component (Table 2).

**Table 2:** Correlations between the individual task variables and the endophenotypic construct

		first-borns		second-borns	
**Task**	**Measure**	*z*-scores	raw data	*z*-scores	raw data
Stop task	Stop signal reaction time	.66	.63	.67	.64
Shifting attentional set	% errors	.63	.67	.67	.71
Time reproduction	Absolute deviation	.70	.59	.74	.67
Visuo-spatial sequencing	*N *identified targets correct order	.73	.72	.79	.80
Digit span	*N *backwards	.55	.53	.69	.69
Pursuit	Precision	.74	.64	.77	.65
Tracking	Precision	.60	.55	.54	.48
Tapping	Variability	.46	.50	.49	.47
Baseline speed	Variability	.52	.48	.70	.62
Motor timing	Variability	.71	.56	.73	.52

### First aim: Predictive validity of the endophenotypic construct for the ADHD diagnosis

A multiple discriminant analysis was performed with age and the endophenotypic construct as predictors and diagnosis as the grouping variable. To correct for the unequal group sizes, prior probabilities for all groups were set to 1/3. The endophenotypic construct significantly predicted diagnostic status (first-borns *F *(2, 328) = 33.24, *p *< .001; second-borns *F *(2, 320) = 29.73, *p *< .001). In all groups, correct classification percentages were roughly around 50%. In the first-borns, respectively 55%, 52%, and 48% of the affected children, non-affected siblings, and controls were correctly classified. In the second-borns, respectively 67%, 41%, and 45% of the affected children, non-affected siblings, and controls were correctly classified.

### Second aim: Group differences at an endophenotypic and phenotypic level

**Figure 3:**
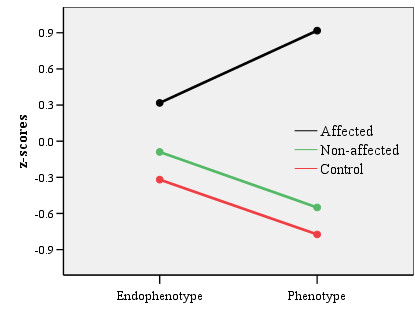
Group differences at the endophenotypic level (operationalized as a composite score of ten task variables) and at the phenotypic level (operationalized as a composite of parental and teacher ADHD questionnaire).

The group by level interaction was analyzed to assess whether group differences were comparable at the endophenotypic and phenotypic level. This interaction was significant (first-borns *F *(2, 327) = 45.46, *p *< .001, η_*p*_^2 ^= .22; second-borns *F *(2, 319) = 56.89, *p *< .001, η_*p*_^2 ^= .26), suggesting group contrasts to be different at the endophenotypical and phenotypical level. When the analysis was repeated with affected children and controls, the interaction remained significant (first-borns *F *(1, 260) = 67.68, *p *< .001, η_*p*_^2 ^= .21; second-borns *F *(1, 242) = 92.13, *p *< .001, η_*p*_^2 ^= .28). As is visible in Figure 3, affected children deviated more from controls at the phenotypic level than at the endophenotypic level. No such interaction was present for non-affected siblings compared to controls (first-borns *F *(1, 185) = 0.24, *p *= .63, η_*p*_^2 ^< .01; second-borns *F *(1, 188) = 0.02, *p *= .89, η_*p*_^2 ^< .01).

### Third aim: Moderating and mediating effects of gender, age, and IQ and the influence of rater bias on the relation between endophenotypic construct and phenotype

#### Gender

No evidence was found for a moderating effect of gender (first-borns *t *= -.34, *β *= -.05, *p *= .73; second-borns *t *= -.41, *β *= -.06, *p *= .69), which suggested that the relation between the endophenotypic construct and phenotype was comparable for boys and girls. There was evidence, however, for a partially mediating effect of gender in the first-borns (Table 3). Part of the relationship between the endophenotypic construct and ADHD was related to gender: Boys performed slightly worse than girls on the endophenotypic construct and boys had more severe ADHD phenotypes than girls.

**Table 3:** Mediating effects of gender, age, and IQ on the relation between the endophenotypic construct and phenotype

**mediator**	endophenotype → mediator			mediator → phenotype			endophenotype → phenotype			test of mediation: *z *= *a *× *b*/√(*b*^2^*s*_*a*_^2 ^+ *a*^2^*s*_*b*_^2^)		endophenotype → phenotype corrected for mediation		
	*a*	*s*_*a*_	*p*	*b*	*s*_*b*_	*p*	*c*	*s*_*c*_	*p*	*z*	*p*	*c*	*s*_*c*_	*p*
**gender**														
first-borns	-0.129	.026	<.001	-0.319	.108	.003	7.215	.657	<.001	2.538	.01	5.963	0.629	<.001
second-borns	-0.020	.028	.47	-0.285	.113	.01	2.390	.771	.002	0.687	.49	2.148	0.697	.002
**age**														
first-borns	-1.724	.117	<.001	-1.383	.271	<.001	7.215	.657	<.001	4.822	<.001	8.041	0.846	<.001
second-borns	-2.130	.102	<.001	0.889	.275	.001	2.390	.771	.002	-3.195	.001	10.150	1.044	<.001
**IQ**														
first-borns	-2.264	.608	<.001	-0.255	.067	<.001	7.215	.657	<.001	2.662	.008	6.916	0.667	<.001
second-borns	-1.222	.633	.05	-0.328	.066	<.001	2.390	.771	.002	1.799	.07	2.012	0.752	.008

#### Age

Age had a moderating effect on the relation between the endophenotypic construct and phenotype in the second-borns (*t *= 2.92, *β *= .58, *p *= .004), though this effect was completely non-significant in the first-borns (*t *= -.02, *β *= -.004, *p *= .99). The significant interaction between the endophenotypic construct and age in predicting ADHD in second-borns appeared to be related to the non-affected siblings: Younger non-affected siblings performed in between their affected siblings and controls (non-affected versus affected *p *< .001 and non-affected versus control *p *= .002), but older non-affected siblings performed more like controls (non-affected versus affected *p *< .001 and non-affected versus control *p *= .54). Strong evidence was found for a partially mediating effect of age: The endophenotypic construct related to age, and age related to ADHD severity (Table 3). Correcting for this partial mediating effect of age did not, however, result in a non-significant direct effect between the endophenotypic construct and phenotype in ADHD.

#### IQ

IQ did not have a moderating influence (first-borns *t *= -1.11, *β *= -.49, *p *= .27; second-borns *t *= -.81, *β *= -.37, *p *= .42), indicating that the relation between the endophenotypic construct and phenotype to be constant across the IQ range studied here. However, there was a partial mediating effect of IQ (Table 3), suggesting the endophenotypic performance related to IQ, and IQ related to ADHD. Correcting for this partial mediating effect of IQ did not result in a non-significant direct route between the endophenotypic construct and phenotype.

#### Rater bias

Paired samples *t*-tests indicated that parents rated the ADHD symptoms of their affected child as more severe than did teachers (first-borns *t *= 4.92, *p *< .001; second-borns *t *= 5.32, *p *< .001), which was in line with our expectations. However, in contrast to our expectations, parents and teachers ratings agreed on the degree of ADHD-like behaviour in non-affected children (first-borns *t *= -.51, *p *= .61; second-borns *t *= .21, *p *= .84), which is similar to the findings in control children (first-borns *t *= .92, *p *= .36; second-borns *t *= .86, *p *= .39). It appeared that parents were indeed possibly 'overestimating' the degree of ADHD in their affected child, but they did not seem to 'underestimate' the degree of ADHD in their non-affected child.

None of the correlation coefficients between the endophenotypic construct and ADHD rated by parents, on the one hand, and the endophenotypic construct and ADHD rated by teachers, on the other hand, differed significantly from each other (*t*-values between 0.47 – 2.47 and *p*-values between .36 – .98), when calculated separately for affected, non-affected, and control children or when calculated across groups. These findings suggest that the predictive validity of the endophenotypic construct was comparable for the phenotype as rated by the parents or by the teachers.

## Discussion

This study aimed at examining the predictive validity of endophenotypic functioning for the ADHD diagnosis, the magnitude of group differences at an endophenotypic and phenotypic level, and the mediating and moderating effects of gender, age, and IQ as well as the influence of rater bias on the relation between endophenotype and phenotype. Analyses were separately conducted on first- and second-borns to accommodate for the non-independency of data. First- and second-borns did not differ with respect to proportion of affected, non-affected and control children, and results were largely consistent across first- and second-borns, suggesting ADHD was not related to birth-order as has been previously observed [70].

Concerning the first aim of our study: The endophenotypic construct significantly predicted the diagnostic status (affected, non-affected, and control), which is in line with the aetiology of psychiatric disorders, in which endophenotypic vulnerabilities lead to phenotypic symptoms. The status of all groups was predicted with roughly similar percentages (around 50%). However, these percentages were only moderate and not high, indicating a substantial overlap in endophenotypic functioning of children with different diagnostic status. Thus, even an aggregated component encompassing multiple endophenotypic measures is not a firm predictor of diagnostic status and illustrates the causal heterogeneity of ADHD [13].

In the second aim of our study, it was confirmed that affected children indeed portrayed a more severe ADHD phenotype than one would expect based on their endophenotypic dysfunctioning. This is in line with previous studies on cognitive dysfunctioning in patients with ADHD, in which not all patients portray neurocognitive dysfunctioning and effect sizes are generally modest [71]. Based on previous endophenotypic studies, we expected to find the reverse pattern for non-affected siblings, portraying a less severe phenotype compared to their endophenotypic construct. This appeared not to be the case: The difference between non-affected siblings and controls was comparable for the endophenotypic construct and phenotype. This suggests that endophenotypic dysfunctioning related proportionally to (subtle) phenotypical deviations in non-affected children, but other factors possibly come into play that aggravate the eventually observable phenotypical problems in affected children. These factors may be environmental in nature, such as differences in upbringing and schooling, which apparently had a more positive outcome for the non-affected sibling than for the affected sibling. It may also be that certain phenotypic symptoms may aggravate other phenotypic symptoms, resulting in a disproportionate severe phenotype in affected children in relation to their endophenotype. For example, a child being hyperactive, may become even more hyperactive because his/her inattention during a school-task results in the child leaving his/her seat.

In line with the idea that some factors may influence the relation between endophenotype and phenotype, we analyzed the (moderating and mediating) effects of gender, age, IQ, and rater bias. No evidence was found for a moderating effect of gender, which suggests that the relation between the endophenotypic construct and phenotype in ADHD is comparable for boys and girls. There was evidence, though, for a partially mediating effect of gender in the first-borns. However, since this partial mediation was completely non-significant in the second-borns, and the direct effect between the endophenotypic construct and phenotype was still present after correcting for this partially mediating effect of gender, it appears that gender did not have a large impact on the relation between the endophenotypic construct and phenotype in ADHD. It could not account for the apparent disproportionately severe phenotype in affected children. Similar non-influential effects of gender in relation to ADHD have been documented previously [72,73].

Another factor possibly influencing the endophenotypic construct – phenotype relation is age. Age moderated the relation between the endophenotypic construct and phenotype in the second-borns, though not in the first-borns. This significant interaction between the endophenotypic construct and age in predicting the ADHD phenotype appeared to be related to the non-affected siblings: Younger non-affected siblings performed in between their affected siblings and controls, but older non-affected siblings performed more like controls, possibly suggesting endophenotypic vulnerabilities ease somewhat with increasing age in non-affected siblings. Strong evidence was found for a partial mediating relation of age, but correcting for this partially mediating effect of age did not result in a non-significant direct effect between the endophenotypic construct and phenotype. The findings of non-moderation of age in affected children are in line with studies showing symptoms of ADHD persist into adolescence and adulthood for the majority of patients [40,74] and suggest that the effect of age does not appear to contribute to the group differences at the endophenotypic and phenotypic levels.

Like gender and age, IQ also did not appear to account for these non-comparable group differences, since IQ did not have a moderating influence on the relationship between the endophenotypic construct and phenotype. IQ did have a mediating effect, but correcting for this mediating effect did not result a non-significant direct route between the endophenotypic construct and phenotype, suggesting the influence of IQ on this relation is not substantial. These findings give support to the idea that endophenotypic dysfunctioning and a lower IQ are both related to each other and to ADHD [47-49], though a lower IQ can not account for the endophenotypic dysfunctioning associated with ADHD.

Another factor we studied was the effect of rater bias on the relation between the endophenotypic construct and phenotype. We hypothesized that parents, compared to teachers, may possibly overrate the ADHD severity in their affected children, whereas they may underrate symptoms in their non-affected children. This, in turn, might explain the disproportionate severe phenotype of the affected group compared to their endophenotypic construct. Parents indeed rated the degree of ADHD in their affected children more severely than teachers. This may be because parents wanted to secure their participation in the study [75] and/or because the problem behaviour was genuinely (experienced as) more severe at home than at school, possibly because the use of symptom reducing medication is more consistent and/or effective at school [75,76]. Either way, although parents did rate the severity of their child's ADHD as more serious than teachers, a possible effect of rater bias could not explain the larger phenotypic dysfunctions compared to endophenotypic dysfunctions in affected children, because the endophenotypic construct related comparably to the phenotype when rated by parents and teachers. Furthermore, no evidence was found for an underestimation of ADHD by parents in their non-affected siblings. Therefore, possible differences in parental and teacher ratings of ADHD do not underlie the disproportionate severe phenotype compared to endophenotype in children with ADHD.

## Limitations

Several possible limitations of this study warrant consideration. Dividing the sample in first- and second-borns doubled the number of statistical tests that were performed. However, we corrected for multiple testing and this approach gave the opportunity to investigate replicability of the results and showed that almost all findings were comparable in first- and second-borns. Another possible limitation was the interpretability of the endophenotypic component measure. Since it was a composition of various cognitive and motor task variables, its exact representation remains unclear. It may be hypothesized that the endophenotypic construct taps into an underlying factor, which may represent general cognitive functioning ('g') possibly in combination with variability of reacting. We feel though, that combining individual task measures to one more robust component will facilitate heritability research in ADHD, since a component probably entails less error variance and may be a more reliable measure than individual task measures.

## Conclusion

An endophenotypic construct encompassing multiple endophenotypic measures is moderately predictive of diagnostic status, but substantial overlap exists between endophenotypic functioning in the groups of affected children, non-affected siblings and controls. Group differences at an endophenotypic and phenotypic level are not comparable for affected children, displaying a more severe phenotype than one would expect based on their endophenotype when compared to controls. Group differences were comparable for non-affected siblings compared to controls, suggesting subtle endophenotypic vulnerabilities translate proportionally into phenotypic deviations. Even though a potentially moderating effect (age) and several mediating effects (gender, age, IQ) have been found affecting the relation between the endophenotypic construct and phenotype, none of the effects studied (gender, age, IQ, and rater bias) could account for the finding that affected children deviated more from controls at the phenotypic than endophenotypic level. These findings suggest other factors come into play and aggravate the phenotype in affected children.

## Abbreviations

ADHD = Attention-Deficit/Hyperactivity Disorder; DSM-IV = Diagnostic and Statistical Manual for Mental Disorders 4^th ^edition; IMAGE = International Multicenter ADHD Genes study; IQ = Intelligence Quotient; PACS = Parental Account of Children's Symptoms; SCQ = Social Communication Questionnaire; SDQ = Strengths and Difficulties Questionnaires; SSRT = Stop Signal Reaction Time.

## Competing interests

NR, MA, NM, CB, and JO have no competing interests. SF has received grants from Eli Lilly, McNeil Consumer & Specialty Pharmaceuticals, and Shire; has been a consultant for Eli Lilly, McNeil Consumer & Specialty Pharmaceuticals, Shire, Noven Pharmaceuticals, and Cephalon; has been on the speakers' bureaus of Eli Lilly, McNeil Consumer & Specialty Pharmaceuticals, Shire, and Cephalon. JB has been a consultant to/member of advisory board of/and/or speaker for Janssen Cilag BV, Eli Lilly, Bristol-Myer Squibb, UBC, Shire, Medice. JS has been a member of advisory board of Eli Lilly, Shire, Janssen Cilag. This study was partly funded by a grant assigned to SF by the National Institute of Mental Health (NIH grant # R01 MH62873-01A1).

## Authors' contributions

NR contributed to the data ascertainment, study design, literature searches, analyses, and writing. MA and CB contributed to the data ascertainment and writing. NM contributed to the analyses and writing. SF, JB, JS and JO have written the study protocol and have contributed to the writing. All authors have read and approved the final manuscript.
